# Pulmonary Talaromycosis: A Window into the Immunopathogenesis of an Endemic Mycosis

**DOI:** 10.1007/s11046-021-00570-0

**Published:** 2021-07-06

**Authors:** Shanti Narayanasamy, John Dougherty, H. Rogier van Doorn, Thuy Le

**Affiliations:** 1 Division of Infectious Diseases and International Health, Duke University School of Medicine, Durham, NC USA; 2 Oxford University Clinical Research Unit, Hanoi, Vietnam; 3 Centre for Tropical Medicine and Global Health, Nuffield Department of Medicine, University of Oxford, Oxford, UK

**Keywords:** *Talaromyces marneffei*, Talaromycosis, Respiratory tract, Cavitation, Nodule, Effusion

## Abstract

Talaromycosis is an invasive mycosis caused by the thermally dimorphic saprophytic fungus *Talaromyces marneffei* (Tm) endemic in Asia. Like other endemic mycoses, talaromycosis occurs predominantly in immunocompromised and, to a lesser extent, immunocompetent hosts. The lungs are the primary portal of entry, and pulmonary manifestations provide a window into the immunopathogenesis of talaromycosis. Failure of alveolar macrophages to destroy Tm results in reticuloendothelial system dissemination and multi-organ disease. Primary or secondary immune defects that reduce CD4^+^ T cells, INF-γ, IL-12, and IL-17 functions, such as HIV infection, anti-interferon-γ autoantibodies, STAT-1 and STAT-3 mutations, and CD40 ligand deficiency, highlight the central roles of Th1 and Th17 effector cells in the control of Tm infection. Both upper and lower respiratory infections can manifest as localised or disseminated disease. Upper respiratory disease appears unique to talaromycosis, presenting with oropharyngeal lesions and obstructive tracheobronchial masses. Lower respiratory disease is protean, including alveolar consolidation, solitary or multiple nodules, mediastinal lymphadenopathy, cavitary disease, and pleural effusion. Structural lung disease such as chronic obstructive pulmonary disease is an emerging risk factor in immunocompetent hosts. Mortality, up to 55%, is driven by delayed or missed diagnosis. Rapid, non-culture-based diagnostics including antigen and PCR assays are shown to be superior to blood culture for diagnosis, but still require rigorous clinical validation and commercialisation. Our current understanding of acute pulmonary infections is limited by the lack of an antibody test. Such a tool is expected to unveil a larger disease burden and wider clinical spectrum of talaromycosis.

## Introduction

Talaromycosis is an invasive mycosis caused by the thermally dimorphic fungus *Talaromyces marneffei* (Tm) endemic to Southeast Asia, southern China, and north-eastern India. Across the endemic region, talaromycosis is estimated to have a pooled prevalence of 3.6% (range 0.13–19.63%) in people living with HIV/AIDS, with the greatest risk of infection in people with a CD4 cell count < 200 cells/mm^3^ (OR 12.68, 95%CI: 9.58–16.77) [1]. Increasingly, talaromycosis has been described in non-HIV-infected people, accounting for 12.3% (82/668) of cases in one estimate [2]. The majority of these cases have other immunosuppressing conditions, including primary immunodeficiencies, auto-immune diseases, malignancies, and iatrogenic immunosuppression [3]. Despite antifungal therapy mortality remains unacceptably high, between 15 and 55% [2, 4–6]. The soil-burrowing bamboo rats are the enzootic reservoir of Tm. However, human talaromycosis is not linked to bamboo rat exposure or consumption; rather, occupational exposure to crops and livestock is a risk factor [7]. Incidence increases 30–50% during the rainy months [6, 8], but is associated with increased humidity, rather than precipitation [9]. This suggests that humidity creates favourable conditions for fungal growth and aerosol dispersion, increasing respiratory exposure and infections in humans. This paper focuses on the immunopathogenesis and pulmonary manifestations of talaromycosis, highlighting unique clinical features, challenges, and research needs.

## Immunopathogenesis

The presence of upper and lower respiratory tract diseases in humans suggests that inhalation is the dominant portal of entry in talaromycosis. Studies in wild bamboo rats have found the highest burden of disease in the lungs (83.3%), followed by the liver (33.3%) and spleen (33.3%) [10]. A murine inhalation model delivering nebulised Tm conidia in a chamber to mice demonstrated that the lungs were the primary organ of infection with invasive pulmonary disease, occurring in 65% of exposed mice [11]. At the host–pathogen interface, Tm conidia adhere to bronchial epithelial cells by attaching to extra-cellular matrix laminin and are phagocytosed by pulmonary alveolar macrophages [12]. Although phagocytosis occurs, Tm evades macrophage killing by producing superoxide dismutase and catalase-peroxidase to prevent digestion by lysosomes [13]. Tm further evades the host defence through down-regulation of IL-6, a pro-inflammatory cytokine produced by bronchial epithelial cells [14], and through the galactomannanprotein Mp1p which effectively captures arachidonic acid and disrupts the host pro-inflammatory cascade [15]. The establishment and proliferation of infection inside macrophages enables Tm to disseminate through the reticuloendothelial system causing multi-organ disease.

Defects in cellular immunity and CD4^+^ lymphopenia are the major predisposing factors for talaromycosis, as demonstrated by the high burden of infection in individuals with advanced HIV and other T cell immunosuppression [3]. Development of talaromycosis among individuals with inborn errors of immunity provides insights into the immune mechanisms essential for disease control. The adult-onset immunodeficiency condition due to anti-interferon-γ autoantibodies inhibits signal transducer and activator of transcription 1 (STAT1) phosphorylation and IL-12 production, leading to a severely compromised Th1 response [16]. In addition to fungal diseases, these individuals are at risk for mycobacterial diseases, disseminated varicella zoster, and salmonellosis [17]. Isolated STAT1 deficiency, both inherited and sporadic, has been described, as well as hyper-IgE (Job’s) syndrome caused by STAT3 mutations resulting in Th-17 deficiency [18]. CD40 ligand deficiency (hyper-IgM syndrome) causes down-regulation of activated *T* cells, resulting in decreased signalling through NF-κB and reduction in IL-12 production [18]. These immune deficiencies reveal the central roles of IL-12 and INF-γ interaction for macrophage activation and Th17 effector cells in host control of Tm.

## Pulmonary Manifestations

Both immunocompetent and immunocompromised individuals can develop talaromycosis, and disease can be localised or disseminated [19]. Disseminated disease has traditionally been associated with advanced HIV infection and localised disease in non-HIV-infected hosts. However, evidence is emerging that both localised respiratory and disseminated disease can develop in HIV-infected and non-HIV-infected hosts and host immune function cannot be inferred from clinical presentation [3, 17, 19, 20]. We will describe the pathology in the lungs based on anatomical location—upper respiratory and lower respiratory tracts—which have been associated with localised as well as disseminated disease.

### Upper Respiratory Tract Infection

**Fig. 1:**
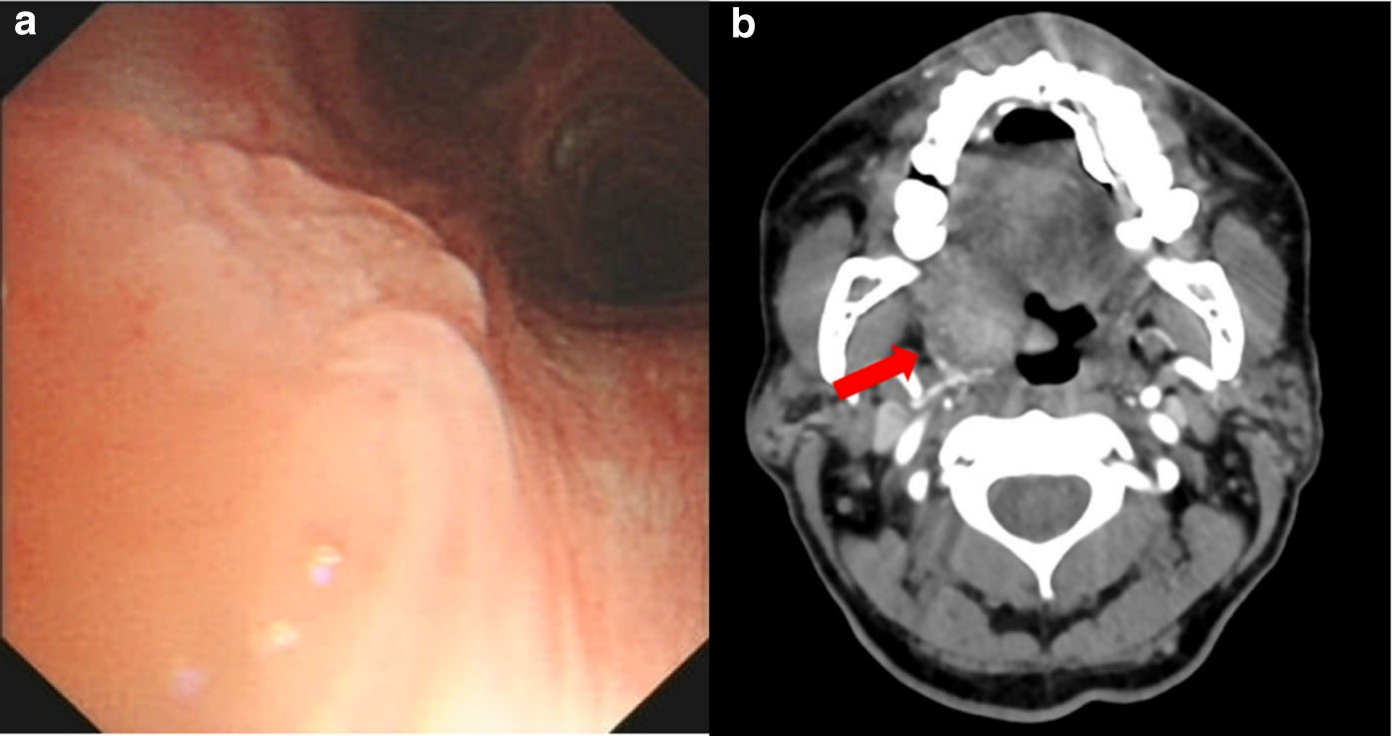
Upper respiratory tract manifestations of talaromycosis. **a** Bronchoscopy demonstrating irregular bronchial mucosal surface, in a 34-year-old immunocompromised female with a STAT3 mutation. Tm was grown from biopsied tissue [59]. **b** Computed tomography (CT) angiogram of the neck demonstrating an ill-defined mass along the right lateral aspect of the hypopharynx involving the base of the tongue, right lingual tonsil, and right vallecula extending along the right palatine tonsil and into the pharyngeal space, in a 63 year-old man with HIV [27]

Uncommon among endemic fungi, upper respiratory tract infection is a unique manifestation of talaromycosis [21] and has been described in immunocompetent and immunocompromised patients [22–25]. Pharyngeal and laryngeal lesions have been identified in patients presenting with odynophagia, dysphagia, hoarseness, soft tissue masses, papules, and mucosal ulceration (Fig. 1) [25–28]. Tracheal and endobronchial lesions are often accompanied by cervical lymphadenopathy, pulmonary infiltrates, or post-obstructive pneumonia [29, 30]. Although uncommon, clinical manifestations can be dramatic with structural collapse of the large airways or tracheal stenosis causing airway obstruction [29, 31]. Tracheobronchial infections can lead to long-term sequelae, requiring tracheostomy and reconstructive surgery [24]. Biopsy for histopathology and cultures for both fungi and mycobacteria are critical to differentiate talaromycosis from tuberculosis, head and neck cancer, lymphoma, or Kaposi’s sarcoma.

### Lower Respiratory Tract Infection

**Fig. 2:**
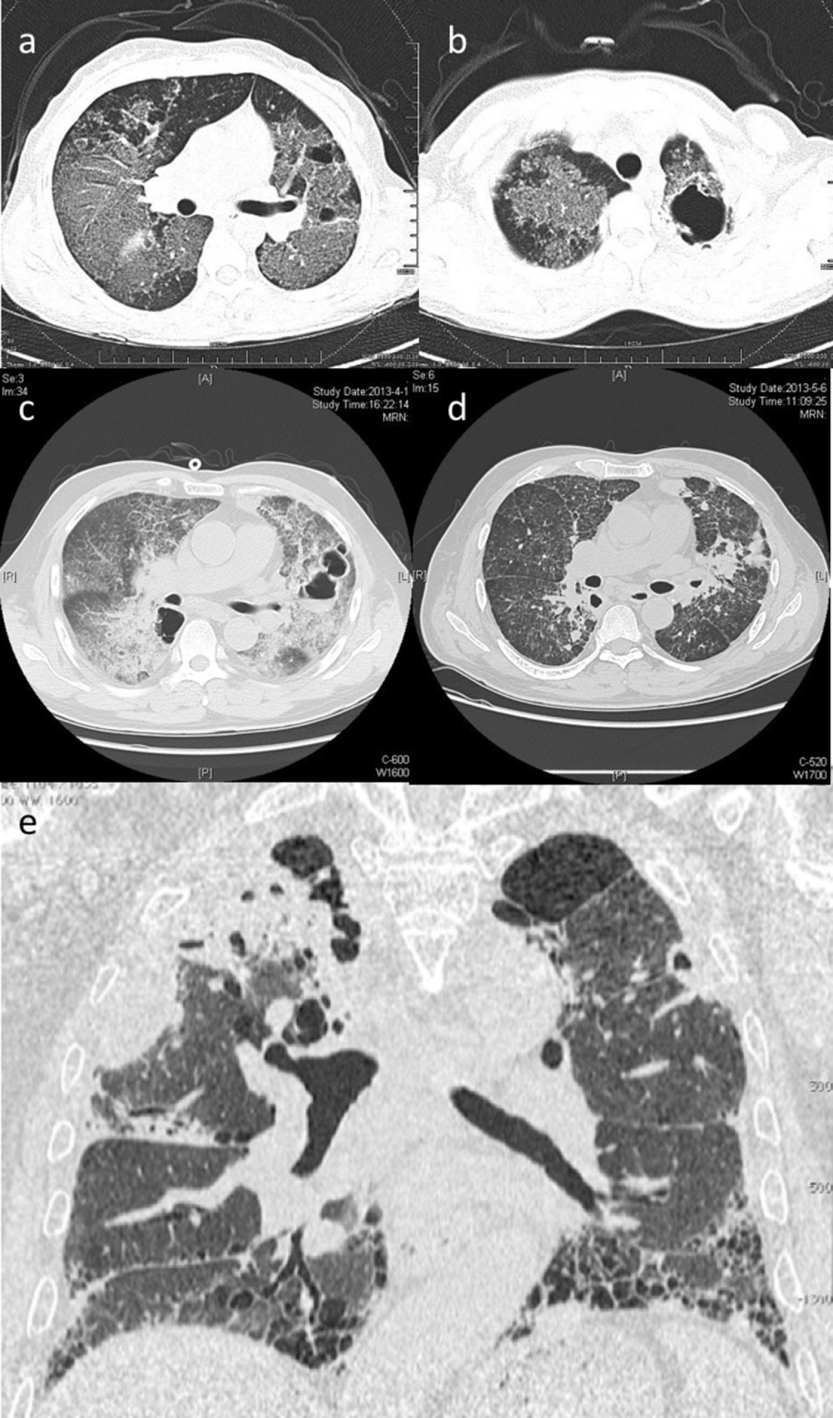
Lower respiratory tract manifestations of talaromycosis. **a** & **b**: CT Chest demonstrating extensive bilateral ground glass changes and multiple bullae, in a 34-year-old immunocompromised female with a *STAT3* mutation [59]. c: CT Chest with diffuse interstitial infiltrates and multiple cavitary lesions in a 57-year-old man following steroid use [60]. CT Chest **d** following treatment, demonstrating marked regression of interstitial and cavitary lung disease. **e** CT Chest demonstrating right upper lobe infiltration with Tm, bibasilar subpleural reticular changes, traction bronchiectasis and honeycombing in a 71-year-old, immunocompetent patient with idiopathic pulmonary fibrosis [61]

Lower respiratory tract infections are protean, encompassing alveolar consolidation, solitary or multiple nodules, mediastinal lymphadenopathy, pulmonary mycetoma, cavitary disease, and pleural effusion (Fig. 2) [22, 32, 33]. Radiological characteristics are similarly diverse, including thick- and thin-walled cavitations, patchy consolidation, ground glass changes, reticular nodular changes, and hilar and mediastinal lymphadenopathy [20, 34, 35]. Cavitary disease had been thought to predominate in non-HIV-infected patients; however, a study found that talaromycosis was the most common cause of cavitary lung lesions in 81 patients with HIV/AIDS (23.5%), followed by cryptococcosis (13.6%) and tuberculosis (13.6%) [36]. The diagnosis of pulmonary diseases in patients with advanced HIV/AIDS is challenging due to the frequency and clinical mimicry of other infections, including *Pneumocystis jiroveci* pneumonia, tuberculosis, cryptococcosis and histoplasmosis, posing significant diagnostic and therapeutic dilemmas.

Pleural effusion is an under-recognised manifestation of talaromycosis. In a non-HIV-infected cohort from China, 42/61 (69%) had pleural effusions; all cases were initially misdiagnosed as tuberculosis [37]. Compared to tuberculous pleural effusions, talaromycosis effusions have a lower protein content and lymphocyte count, and a higher neutrophil count. The diagnosis is often made by pleural biopsy and culture, and thoracoscopy demonstrates fibrous pleural adhesions and multiple pleural nodules [37]. Talaromycosis may be unique among endemic mycoses and other opportunistic mycoses (i.e. aspergillosis, mucormycosis) in causing pleural effusions and should be considered in the differential diagnosis in a susceptible host [22].

## Structural Lung Disease in Immunocompetent Hosts

Structural lung disease is increasingly recognised as a risk factor for talaromycosis among immunocompetent hosts. Case reports describe lung malignancy, both primary and metastatic, leading to talaromycosis post-obstructive pneumonia or diffuse infiltrates. These cases have been diagnosed prior to any immunosuppression, at cancer presentation [38–40]. Talaromycosis has also been described in immunocompetent patients with chronic obstructive pulmonary disease and disruption of lung architecture from other cavitary diseases [41, 42]. Cases of pneumonia and disseminated disease have been reported in immunocompetent hosts [20, 43, 44], suggesting that talaromycosis may be a more common cause of community-acquired pneumonia than currently recognised. At present, incidence of non-severe disease in immunocompetent hosts is not known, as diagnostic capabilities for talaromycosis are largely restricted to culture, which is poorly sensitive for diagnosis [45].

## Challenges and Advances in Diagnosis and Treatment

Diagnostic delay is the most critical barrier to reducing morbidity and mortality. Aside from the characteristic central-umbilicated skin lesions seen in 50–70% of disseminated disease, talaromycosis has no pathognomonic clinical or radiological syndrome. It shares an endemic area and clinical presentations with tuberculosis, non-tuberculous mycobacteria, histoplasmosis, cryptococcosis, rhodococcus, and melioidosis, often resulting in misdiagnosis as one of these infections, delays in treatment, dissemination of infection, and poor patient outcomes [22, 46, 47]. Diagnostic delay is associated with higher mortality, regardless of immunosuppressive status [2, 22]. Increased access to HIV and antifungal therapy have not improved Tm mortality, suggesting that diagnostic delay remains a significant barrier to survival [4].

A preliminary diagnosis of talaromycosis can be made on microscopic examination of skin scrapings, bone marrow and lymph node aspirate, or tissue section [48]. A definitive diagnosis can be made with histopathologic examination or culture. Bone marrow culture has the highest yield (100%), followed by skin (90%), and blood (70%) [6, 49].

The commercial assay for serum Aspergillus galactomannan was investigated in HIV-positive patients with talaromycosis and found a sensitivity of 95.8% (23/24) and specificity of 90.9% (30/33) at an optical density cut-off index of 1.0 [50]. A more recent study of the same assay found a lower sensitivity of 80.56% (29/36) and a specificity of 90% (27/30) at a lower optical density cut-off index of 0.5 [51]. The β-D-glucan assay has been used in some case reports [52]. These antigen-detection assays may have a role as a screening tool for patients at high risk for talaromycosis and other invasive fungal infections in high burden regions due to their cross-reactivity with other endemic fungal pathogens [52].

Several promising non-culture-based diagnostics specific for talaromycosis are in development. The galactomannanprotein Mp1p is a sensitive and highly specific target for antigen detection by enzyme immunoassay (EIA) [53]. Compared to blood culture, the Mp1p EIA improved time-to-diagnosis from 6.6 ± 3.0 days to 6 hours, and improved sensitivity from 72.8% (271/372) to 86.3% (321/372); higher sensitivity was demonstrated with combined plasma and urine testing [45]. Real-time quantitative PCR assays show promise in those with fungemia (sensitivity 100%, [20/20]) and without fungemia (68.75%, [11/16]) [51], and recent development of the 4D1 monoclonal antibody against the yeast phase of Tm has shown high specificity and sensitivity in an inhibitory-EIA and an immunochromatographic test [54, 55].

There are a lack of studies to guide treatment specifically for pulmonary talaromycosis. Evidence for talaromycosis treatment has largely come from studies in HIV-infected patients with disseminated disease. Induction therapy with amphotericin B for two weeks followed by prolonged periods of consolidation and maintenance therapy with itraconazole is highly effective when combined with suppressive HIV therapy [56]. Optimal treatment for localised disease of the respiratory tract has not been defined. Single-agent itraconazole, voriconazole, and amphotericin B have been used [22, 57]. In a report of two non-HIV-infected patients with disease involving the trachea, relapse occurred in one patient at three months despite systemic antifungal therapy and surgery, whereas cure at one year was observed in the patient who received systemic and adjunctive inhaled amphotericin B for two months, suggesting a role for inhaled amphotericin B in treatment of localised respiratory tract disease [58]. Optimal duration of therapy for non-HIV-infected patients remains to be defined. At present, treatment choice and duration are individualised based on the underlying immunodeficiency state, antifungal and immunosuppressive drug-drug interactions, and the expected timeframe of immune dysfunction.

## Conclusions

Pulmonary talaromycosis is clinically diverse and is likely more common than currently recognised. Unlike other endemic mycoses, talaromycosis has a propensity to cause nodules and masses in the upper respiratory tract, and cause pleural effusions. Lower respiratory disease is increasingly recognised in immunocompetent hosts with structural lung diseases. Delay in diagnosis is the most challenging clinical problem. Mortality remains unacceptably high. Research should focus on improving disease detection through development and rigorous clinical validation of antigen detection and PCR-based assays. Development of antibody testing is needed to diagnose acute pulmonary infections and to enable accurate estimates of disease burden and geographical risk regions. These tools are expected to reveal a higher incidence and a wider clinical spectrum of talaromycosis than currently recognised.
